# Uncovering genetic variation in humoral inborn errors of immunity in African populations: insights from the African genome variation database

**DOI:** 10.1038/s41598-026-39612-2

**Published:** 2026-02-15

**Authors:** Luyanda Hlongwa, Ayton Meintjes, Nicola Mulder, Elizabeth Mayne

**Affiliations:** 1 Division of Immunology, Department of Pathology, Faculty of Health Sciences, University of Cape Town, Cape Town, South Africa; 2 Division of Computational Biology, Department of Integrative Biomedical Sciences, Faculty of Health Sciences, University of Cape Town, Cape Town, South Africa; 3 Faculty of Health Sciences, Institute of Infectious disease and Molecular Medicine, University of Cape Town, Cape Town, South Africa; 4 Department of Immunology, National Health Laboratory Services, Cape Town, South Africa

**Keywords:** Computational biology and bioinformatics, Diseases, Genetics, Immunology

## Abstract

Inborn Errors of Immunity (IEIs) are rare genetic disorders affecting immune function. Humoral IEIs, the most commonly diagnosed subtype, are characterised by antibody deficiencies. There is limited data regarding the variation of genes associated with humoral IEI in Africa with implications for designing genetic assays for testing for these diseases. This study aimed to assess the genetic variation in genes which are commonly associated with humoral IEI in African populations. Using the African Genome Variation Database (AGVD) which contain genotype frequencies from African populations. We analysed genotype frequency data from the African Genome Variation Database (AGVD), covering diverse African populations, to identify variants in 23 genes associated with humoral IEIs. Variants were annotated using Ensembl’s Variant Effect Predictor and classified for clinical significance with ClinVar. For variants absent in ClinVar, predictive tools (SIFT, PolyPhen-2) were used. A total of 815 variants were identified; 335 were present in African populations, and 219 were unique to African populations. Most were missense mutations, 4 variants identified in TCF3 and RAG1/2 were classified as pathogenic or likely pathogenic. Additionally, 144 variants (43%) were not listed in ClinVar; of these, 53 were predicted to be deleterious. Some variants exhibited high genotype frequencies (e.g., up to 0.67 in CR2), suggesting possible adaptive functions or population-specific relevance.

**Supplementary Information:**

The online version contains supplementary material available at 10.1038/s41598-026-39612-2.

## Introduction

Inborn Errors of Immunity (IEIs), also known as primary immunodeficiencies, are disorders caused by genetic defects affecting either the innate or adaptive immune function or both^1,2^. Clinically, IEIs present mainly as recurrent or severe infections, immune dysregulation (autoimmune or autoinflammatory disorders), and lymphoproliferation with or without dysmorphic features^3^. IEIs are often misdiagnosed or diagnosed only after the development of serious or life-threatening complications^4^. The prevalence of IEIs is still poorly understood for several reasons, including under-diagnosis, under-reporting, and lack of physician awareness. Although IEIs are considered rare^5^, systematic reviews of IEIs patient registries suggest that IEIs may be present in as many as 1:5000 to 1:1000 live births^6^. In Africa, one million individuals are estimated to be suffering from IEIs, with nearly 42 000 of these cases in South Africa^7,8^. The commonest disorders reported in South Africa are humoral IEIs, characterised by antibody deficiencies^6^. In low- and middle-income countries (LMIC) such as South Africa, there is a lack of diagnostic capacity for IEIs diagnosis, which is further compounded by the high infectious disease burden^9^. Diagnostic testing in South Africa, including sequencing for causative mutations, is not widely available especially in the State sector.

There are 485 individual IEIs as of 2022 which are primarily identified by the underlying genetic abnormality^10^. The International Union of Immunological Societies (IUIS) classify IEIs according to the different components of the immune system involved. Humoral IEIs, also called primary antibody deficiencies, are a sub-group characterised by impaired antibody production^11^ and represent nearly 70% of all diagnosed IEIs with a global prevalence of 1:100 000–1:8 500^6^ although in certain populations and for certain disorders the prevalence may be higher^12,13^. Patients with IEIs may present with recurrent infections, autoimmunity, autoinflammation and lymphoid malignancies which may complicate the diagnosis^14^ by masking infection. The severity of humoral IEIs varies which may present with isolated immunoglobulin subclass deficiencies or with absent or virtually absent B-cell responses. Patients with severe humoral IEIs require life-long immunoglobulin replacement therapy and in some cases prophylactic antibiotic treatment^14^.

**Table 1:** Humoral IEI with commonly affected genes^10^.

Condition	Gene	Function	OMIM number
Agammaglobulinemia	IGHM	Constant region of immunoglobulin mu-chain	147020
	IGLL1	One of the surrogate chains forming the pre-B cell receptor, important for the development of B cells from pro-B cells to pre-B cells.	146770
	BLNK	Cytoplasmic linker important in B-cell development	604515
	BTK	Plays a crucial role in B cell development	300300
	CD79A	Ig-alpha, part of the B cell receptor. Important for B cell development	112205
	CD79B	Ig-beta, part of the B cell receptor. Important for B cell development.	147245
	PAX5	B cell-specific activator protein, a transcription factor binding to promoters of the CD19 gene	167414
	PIK3CD	Class 1 PIK3 protein, which binds to p85 proteins and GPT-bound RAS	602839
	PIK3R1	85 kDa subunit of the phosphatidylinositol 3-kinase enzyme	171833
	SLC39A7	Responsible for transporting Zinc from the Golgi and reticulum to the cytoplasm. Important for the activation of tyrosine kinases.	601416
	SPI1	ETS domain transcription factor which activates gene expression during myeloid and B cell development.	165170
	TCF3	Two basic helix-loop-helix transcription factors (E12 & E47) involved in regulation of immunoglobulin gene expression	147141
	TOP2B	DNA topoisomerase II, an enzyme that relieves topological stress during DNA replication.	126431
	LRRC8A	Facilitates the transport of cyclic GMP-AMP across membranes, binds identical proteins, and functions as a volume-regulated ion channel.	608360
	FNIP1	The protein is involved in the regulation of cellular metabolism and nutrient sensing by influencing AMPK and mTOR signalling pathways.	
Common variable immunodeficiency (CVID)	ARHGEF1	Member of the guanine nucleotide exchange factor family, specific for GTPase RhoA	601855
	ATP6AP1	Subunit of the vacuolar (V)-ATPase protein pump	300197
	CD19	Member of the immunoglobulin superfamily, earliest marker of B cells.	107265
	CD20	B cell surface marker that plays a role in the differentiation of B cells into plasma cells.	112210
	CD21 (CR2)	Receptor of complement component C3d, EBV binds to this receptor during infection.	120650
	CD81	Member of the transmembrane 4 superfamily, which mediates signal transduction events that regulate cell development, activation, growth and motility.	186845
	IKZF1	Transcription factor of the zinc finger family, DNA-binding proteins associated with chromatin remodelling	603023
	IRF2BP2	Protein that interacts with the C-terminal of the transcriptional repression domain of IRF2	615332
	KARS1	Lysyl-trna synthase, which catalyses the aminoacylation of trna-lys in both the cytoplasm and mitochondria	601421
	MOGS	First enzyme of the N-linked oligosaccharide processing pathway.	601336
	NFkB1	Precursor of the DNA-binding subunit of the transcription regulator NFkB	164011
	NFkB2	Subunit of the transcription complex, NFkB, can be an activator or a repressor, depending on the dimerisation partner	615577
	PIK3CD	Class 1 PIK3 protein, binds to p85 proteins and GPT-bound RAS	602839
	PIK3R1	Codes for the 85 kDa subunit of the phosphatidylinositol 3-kinase enzyme	171833
	POU2AF1	Involved in positive regulation of transcription by RNA polymerase II	601206
	PTEN	Phosphatidylinositol-3,4,5-triphosphate-3-phosphatase, dephosphorylates phosphoinositide substrates.	601728
	RAC2	GTPase belonging to the RHO family induce the interferon gamma promoter.	
	TNFRSF13B	Lymphocyte-specific member of the tumour necrosis factor receptor family, induces activation of transcription factors NFAT, AP1, NFkB	604907
	TNFRSF13C	B cell activating factor receptor involved in B cell survival and development	606269
	TNFSF12	Cytokine belonging to the TNF ligand family	602695
	TRNT1	CCA-adding enzyme belonging to the tRNA nucleotidyl transferase/ Poly(A) polymerase family	612907
	CTLA4	Part of the immunoglobulin superfamily whose function is transmitting inhibitory signals to T cells.	123890
	ICOS	From homodimers and functions in cell to cell signalling, immune responses, and regulation of cell proliferation.	604558
	IL21	Plays a role in the immune system by inducing differentiation, proliferation, and activity of multiple immune cell targets.	605384
	IL21R	Receptor to IL21 cytokine, transduces growth promoting signals and functions in the proliferation and differentiation of T, B and NK cells.	605383
	LRBA	Involved in the formation intracellular vesicles to activated receptor complexes.	606453
	MS4A1	Functions in the development and differentiation of B cells into plasma cells.	112210
	PRKCD	Involved in B cell-mediated signalling.	176977
	PLCG2	Plays a role in transmitting signals from growth factor receptors and immune system receptors across the cell membrane.	600220
	SEC61A1	Essential for insertion of secretory and membrane proteins into the endoplasmic reticulum.	609213
	VAV1	Important in haematopoiesis, playing a role in the development and activation of T and B cells.	164875
	BLK	Plays a role in B cell receptor signalling and the development of B cells.	191305
	ZBTB20	Functions as a transcriptional repressor and contributes to processes such as neurogenesis, regulation of glucose levels and postnatal growth.	606025
Hyper IgM syndrome	AICDA	RNA editing deaminase involved in somatic hypermutation, gene conversion and class-recombination.	605257
	CTNNBL1	Component of the pre-mRNA processing factor 19 - cell division cycle 5-like protein complex, activates pre-mRNA splicing. Binds to AICDA.	611537
	INO80	Catalytic ATPase subunit of the INO80 chromatin remodelling complex.	610169
	MSH6	heterodimerize with MSH2 form a complex that exchanges ADP and ATP as DNA mismatches are bound, and dissociate.	600678
	TNFSF13	APRIL, a proliferation-inducing ligand of the TNF ligand superfamily	604472
	UNG	One of the several uracil-DNA glycosylases, prevents mutagenesis by eliminating uracil from DNA molecules	191525
	CD40LG	Protein encoded by this gene is expressed on T cells and regulates B cell function by engaging CD40 on the surface of the B cell.	300386
	CD40	Important in a number of immune responses including T cell-dependent class-switching, memory B cell development and germinal centre formation.	109535
	IKBKG	Regulatory subunit of the IKK complex regulating the activity of nuclear factor kappa B.	300248
Isotype, light chain, or functional deficiencies	CARD11	Protein that acts as a scaffold for NFkB, controlling peripheral B-cell differentiation	607210
	IGKC	Constant region of the kappa light chain of immunoglobulins	147200

Most IEIs are associated with well-characterised genetic mutations (Table 1). Humoral IEIs are linked to abnormal B-cell development which may affect B-cell maturation and B-cell receptor rearrangement. Common variable immunodeficiency (CVID), characterised by deficiencies in antibody isotypes, typically results from gene mutations affecting downstream signalling following B-cell-T-cell interactions. X-linked agammaglobulinemia, the most severe form of humoral IEI, is most commonly caused by mutations in a tyrosine kinase (BTK), which allows signalling through the B-cell receptor following heavy chain rearrangement^15^. Genetic testing is increasingly used to diagnose humoral IEI, although there is significant heterogeneity in underlying abnormalities and the genes which are involved, which may complicate the interpretation of these studies^16^.

In response to the lack of reporting on IEI in Africa, many countries, including South Africa, have established IEI registries. The South African Primary Immunodeficiency Registry (SAPIDR) contains data from 446 individual patients. African patients comprise 17% of the patients in the registry, but 81.4% of the population^17^. Data on gene mutations causing IEIs in South African and other African populations are lacking^17^. It is likely, given genetic diversity in Africa, that disease-causing variants may differ in the African population from populations elsewhere^8^.

In preparation for the development of targeted gene panels to diagnose IEIs in African populations, we aimed to investigate the variation in genes associated with humoral IEI in a database containing genotype frequencies from African populations.

## Methods

### Ethics

 Ethics approval for this project were granted by the Human Research Ethics Committee at the University of Cape Town (Ethics number: 538/2022). Additionally, AGVD has its own ethics approval for analysis of variants from existing aggregate data in target genes (R037/2020). AGVD data were collected from various H3 Africa projects, participants provided informed consent and agreed to data sharing and future use. The University of Cape Town Human Research Ethics Committee provides this ethics. This study was conducted in accordance with the guidelines, including those of the University of Cape Town Human Research Ethics Committee and the Declaration of Helsinki.

A retrospective analysis of data obtained from the African Genome Variation Database (AGVD) (https://nyame.h3abionet.org/accounts/login/?next=/refresh ) of the H3ABioNet Consortium was performed. The AGVD is currently in its beta phase and contains genotype frequency data from individuals representing diverse African populations, compiled from various research projects under the H3Africa initiative^18^. The database provide genotype frequencies for African populations in the following African regions: Central Africa, Eastern Africa, Southern Africa and Western Africa (Table 2)^19^. The data base was made from 1658 samples from individuals of African ancestry and 2552 non-African individuals. The read depth of projects included in the database ranged between 25X to 65X.

**Table 2:** African regions.

Region	Countries
Central Africa	Republic of Burundi, Republic of Cameroon, Central African Republic, Republic of Chad, Republic of the Congo, Democratic Republic of Congo, Republic of Equatorial Guinea, Gabonese Republic, Democratic Republic of São Tomé and Príncipe
Eastern Africa	Union of the Comoros, Republic of Djibouti, State of Eritrea, Federal Democratic Republic of Ethiopia, Republic of Kenya, Republic of Madagascar, Republic of Mauritius, Republic of Rwanda, Republic of Seychelles, Federal Republic of Somalia, Republic of South Sudan, Republic of the Sudan, United Republic of Tanzania, Republic of Uganda
Northern Africa	People’s Democratic Republic of Algeria, Arab Republic of Egypt, Libya, Islamic Republic of Mauritania, Kingdom of Morocco, Sahrawi Arab Democratic Republic, Republic of Tunisia
Southern Africa	Republic of Angola, Republic of Botswana, Kingdom of Eswatini, Kingdom of Lesotho, Republic of Malawi, Republic of Mozambique, Republic of Namibia, Republic of South Africa, Republic of Zambia, Republic of Zimbabwe
Western Africa	Republic of Benin, Burkina Faso, Republic of Cabo Verde, Republic of Côte d’Ivoire, Republic of the Gambia, Republic of Ghana, Republic of Guinea, Republic of Guinea-Bissau, Republic of Liberia, Republic of Mali, Republic of Niger, Federal Republic of Nigeria, Republic of Senegal, Republic of Sierra Leone, Togolese Republic

### Search for variants in humoral IEI-associated genes

 A systematic search was conducted in the AGVD to identify variants in genes commonly associated with humoral IEIs^10^. Each target gene was manually queried using the AGVD search function to retrieve all recorded variants. The resulting variant data were exported to Microsoft Excel for further processing. Variants were annotated using Ensembl VEP (version 112)^20^ by querying rsIDs against the GRCh38 human reference genome. Annotation was performed using MANE Select transcripts where available; if not available, canonical transcripts were used. For downstream analysis, only variants classified as missense, stop-gained, stop-lost, start-lost, or splice-site mutations were retained. Identified variants were categorised based on the population in which they were observed (African or non-African). Variants identified in African populations were further evaluated for clinical significance using the ClinVar database.

### Variant significance

 ClinVar (National Institutes of Health, United States of America, http://www.ncbi.nlm.nih.gov/clinvar/) provides a freely and publicly available archive of many medically significant gene variants. ClinVar clinical significance is recorded as part of the details provided by the VEP tool for each variant where available. Variants identified in the AGVD were classified according to their clinical significance as recorded in ClinVar. ClinVar classifies variants as pathogenic, likely pathogenic, benign, likely benign and of uncertain significance. The terms are described in detail in the guidelines published by the American College of Genetics and Genomics (ACMG) and Association for Molecular Pathology (AMP)^21,22^. Briefly, variants are classified as pathogenic if there is strong evidence to support their association with disease, as likely pathogenic if there is a high probability of them being disease-causing but with lower levels of evidence, as benign if they are not associated with disease and as likely benign if they are unlikely to cause disease. Variants are classified as variants of uncertain significance (VUS) if there is insufficient evidence to classify them as either benign or pathogenic. Using the VEP tool, variants can be classified as deleterious (low/high confidence) or tolerated (low/high confidence) using Sorting Intolerant From Tolerant (SIFT) score (version 6.2.1.)^23^. A SIFT score of 0.0 to 0.05 are predicted to be deleterious with the lowest scores (closest to 0) predicting deleterious effects with high confidence. Variants with a score between 0.05 and 1.0 are predicted to be tolerated with the highest scores (closest to 1.0) predicting tolerated effects with high confidence. Variants can also be classified as possibly damaging, probably damaging or benign using PolyPhen-2^24^.

## Results

**Fig. 1:**
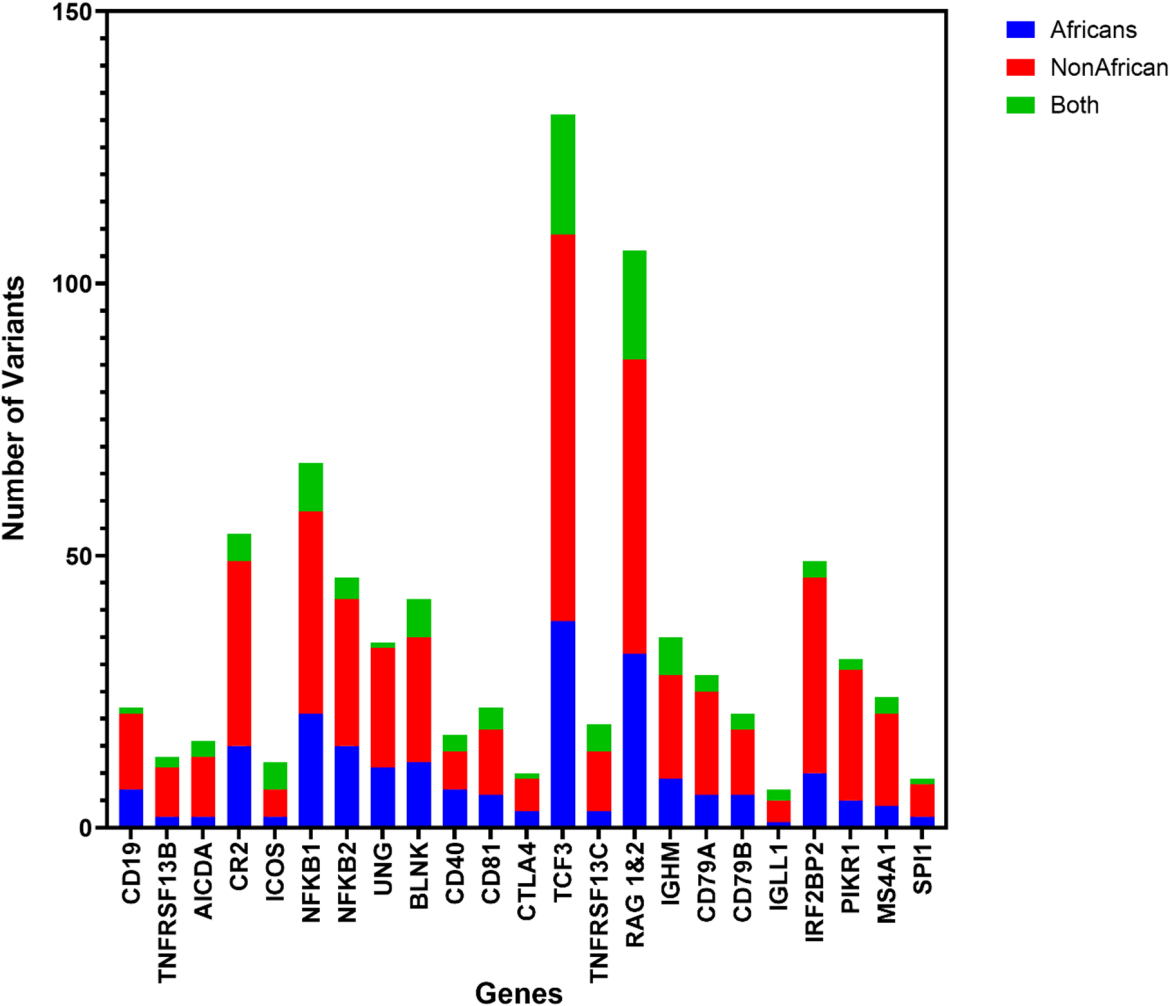
Variant distribution across African and non-African populations. Variants that were identified were grouped according to the population in which they were identified. African populations – 219, non-African populations – 480, identified in both populations – 116.

**Fig. 2:**
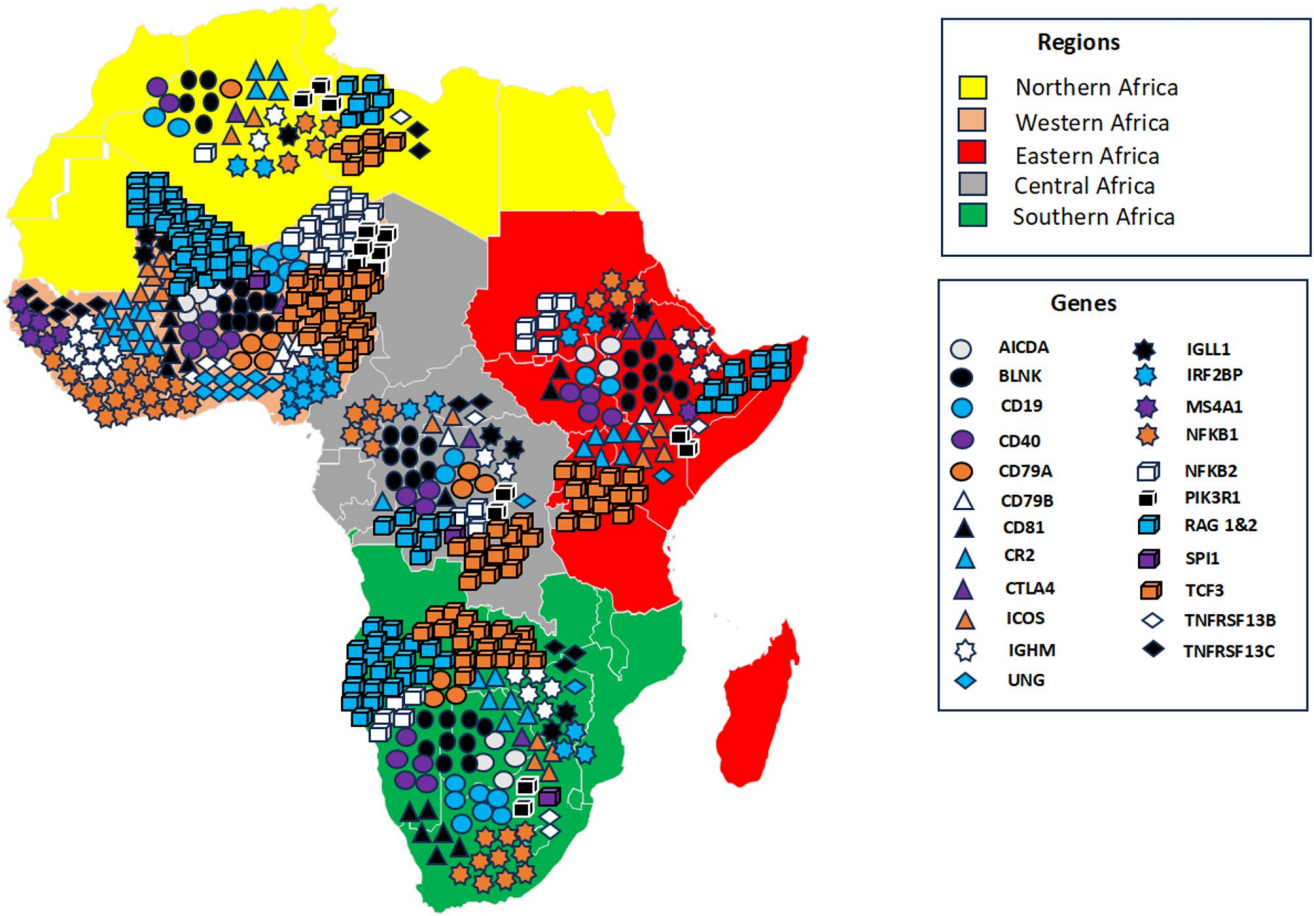
Geographic distribution of gene variants across African regions. Each region is color-coded according to its geographic classification (Northern, Western, Eastern, Central, and Southern Africa). Distinct shapes and colours represent specific genes, as indicated in the legend. The number of symbols displayed within each region corresponds to the number of identified gene variants for that gene in that region.

A systematic search on the AGVD was conducted for variants in genes known to be associated with humoral IEIs. A total of 815 gene variants were identified in 23 genes associated with humoral IEI of which 335 were identified in African populations; 219 gene variants were identified in African populations only, 480 gene variants in Non-African populations only and 116 gene variants identified in both populations (Fig. 1, Supplementary Table S1). The geographic distribution of gene variants across African populations was analysed, revealing distinct regional patterns. Genetic variants were predominantly clustered in Western and Southern Africa, while Northern Africa exhibited comparatively lower genetic diversity (Fig. 2).

**Fig. 3:**
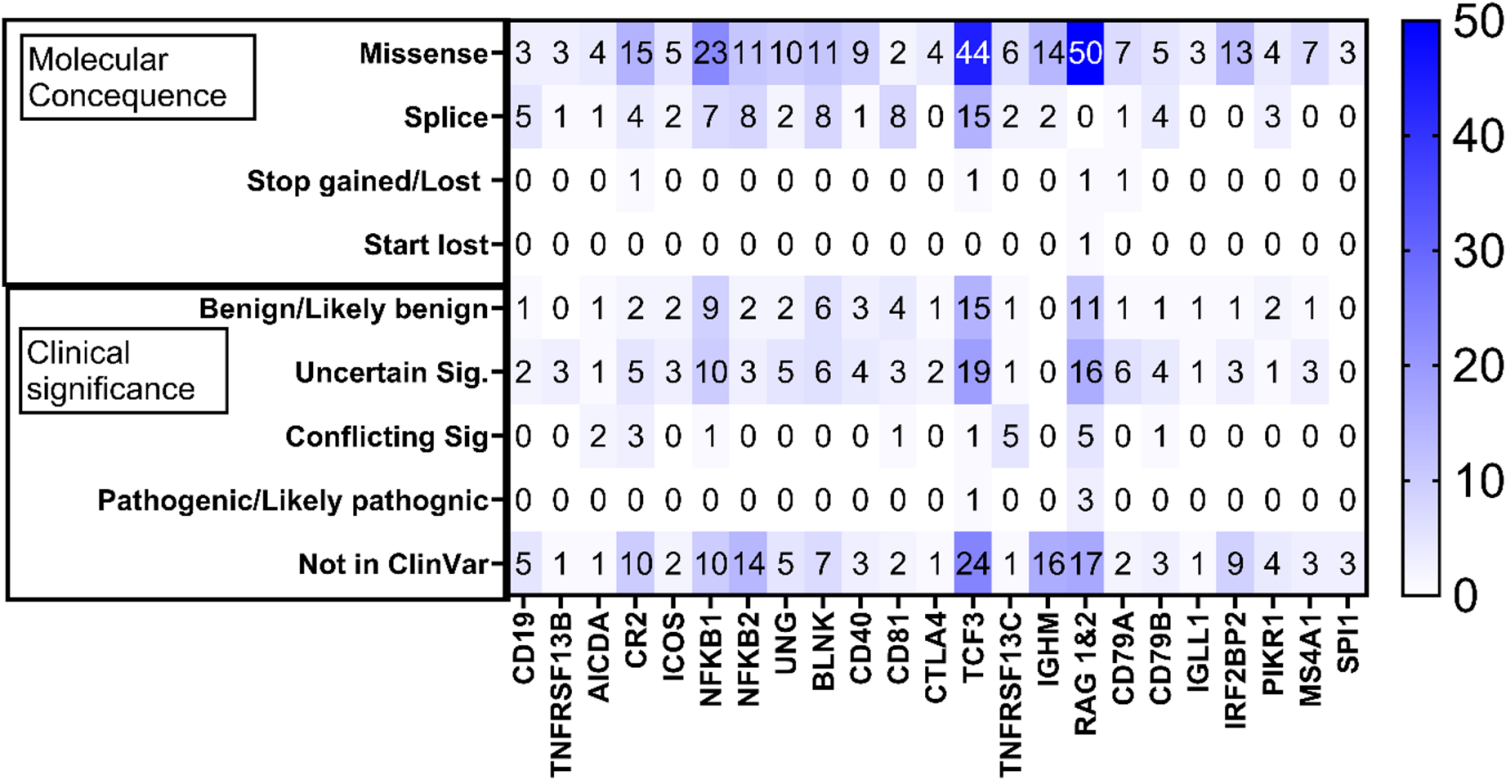
Clinical significance and Molecular consequence. Variants were grouped according to their clinical significance and molecular consequence. There were 4 categories under Molecular consequence. Missense – 256, splice – 74, Stop gain/loss – 4, Start loss – 1. There were 5 categories under clinical significance. Benign/Likely benign – 67, Uncertain significance – 101, Conflicting significance – 19, Pathogenic/likely pathogenic – 4 and Not in ClinVar (for variants not recoded in ClinVar) – 144.

### Molecular consequence and clinical significance

The 335 variants that were identified in the African population, including variants identified in both African and non-African populations were classified according to the predicted molecular consequence. The majority of the variants identified were classified as missense mutations, followed by splice mutations. Only 3 variants (in the CR2, TCF3 and RAG genes) were identified as stop-gain or -loss, while 1 variant (in the RAG gene) was identified as start-lost. Identified variants were classified according to their clinical significance as recorded in ClinVar. Sixty-six (65) variants were classified as benign or likely benign, 95 variants as uncertain, 19 variants as conflicting significance and 4 variants as pathogenic or likely pathogenic (details of pathogenic or likely pathogenic variants supplementary Table S2). Of the variants classified as pathogenic or likely pathogenic, 1 was from the TCF3 gene (frequency of 0.00396 in West African population) and 3 from the RAG 1 & 2 genes (frequency of 0.000495 in West African population) (Fig. 3).

### Variants not in ClinVar

**Fig. 4:**
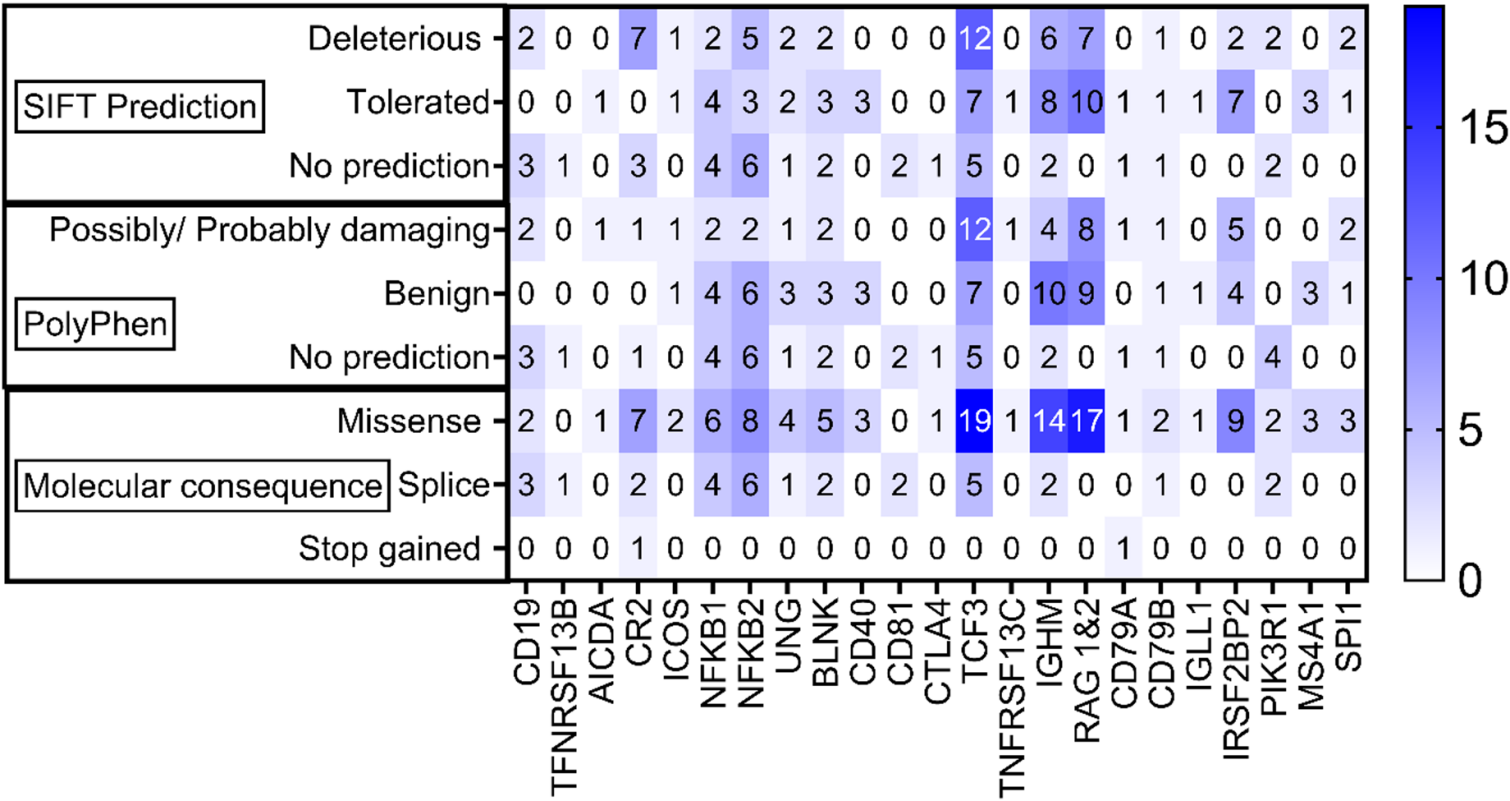
SIFT and PolyPhen-2 predictions and Molecular consequence of variants not recorded in ClinVar. Clinical significance for variants not recorded in ClinVar could not be determined. SIFT predictions, PolyPhen predictions and Molecular consequence were used to assess potential impact of these variants.

 A total of 144 variants from African populations were not recorded in ClinVar (43%). To assess the potential impact of these variants, SIFT and PolyPhen-2 predictions were considered. Of the 144 variants, 53 (36.8%) were predicted to be deleterious, 57 (39.6%) were predicted to be tolerated and 34 (23.6%) were without SIFT predictions. The variants were also grouped according to their PolyPhen-2 predictions as possibly/probably damaging, benign, or no prediction. Of the total Variants, 46 variants were predicted to be possibly/probably damaging, 56 were predicted to be benign, and 34 variants had no PolyPhen-2 predictions. 111 variants were identified as missense mutations, 31 as splice mutations, and 2 as stop-gained mutations. The stop-gained mutations were found in the CR2 and CD79A genes (Fig. 4) with a genotype frequency of 0.000495 in the West African population. The genotype frequency of variants not in ClinVar and predicted to be deleterious by either SIFT or PolyPhen-2 varied in the different African Populations. Genotype frequencies for these variants ranged between 0.076692 and 0.6692 in Central African populations, 0.004237 to 0.5424 in Eastern African populations, 0.0125 to 0.45 in Northern African populations, 0.001866 to 0.4086 in Southern African populations and 0.000495 to 0.5376 in Western African populations. While most of the variants were low frequency variants, several variants had notably high genotype frequencies in different African populations. These include variants in CR2, PIKR3, RAG 1 and 2, and IGHM (Table 3).

**Table 3:** Genotype frequencies of variants not in clinvar predicted to be deleterious.

Variant ID	rsID	Gene	Central Africa	Eastern Africa	Northern Africa	Southern Africa	Western Africa
12_8606914_C_T	rs759799595	AICDA	0	0	0	0	0.000495
10_96216721_A_T	rs587723641	BLNK	0	0	0	0	0.000495
10_96209853_G_A	rs781845656	BLNK	0	0	0	0	0.000495
10_96227501_C_T	rs17111469	BLNK	0.07692	0.0339	0	0.03172	0.01485
16_28937781_C_T	rs200792237	CD19	0	0.004237	0	0	0
16_28937025_C_T	rs113797527	CD19	0.007692	0	0	0.003731	0
19_41879058_G_A	rs557160710	CD79A	0	0	0.0125	0	0
17_63930335_G_A	rs375597674	CD79B	0	0	0	0	0.0009901
1_207468893_A_T	rs368883636	CR2	0	0	0	0	0.000495
1_207454465_C_G	rs375649712	CR2	0	0	0	0.001866	0
1_207469989_A_G	rs551427271	CR2	0	0	0	0	0.0009901
1_207468703_C_G	rs769415969	CR2	0	0	0	0.001866	0
1_207472823_G_T	rs144075435	CR2	0.01538	0.008475	0	0.001866	0.02525
1_207480019_A_G	rs17618	CR2	0.2077	0.02966	0.0625	0.1362	0.07723
1_207473553_T_C	rs4308977	CR2	0.6692	0.5424	0.45	0.4086	0.5376
2_203955686_G_C	rs539709751	ICOS	0	0	0	0	0.000495
2_203936854_C_T	rs77411896	ICOS	0	0.008475	0	0.001866	0.03069
14_105854656_G_A	rs372061764	IGHM	0	0	0	0	0.003521
14_105854644_G_A	rs530414987	IGHM	0	0.004348	0	0	0
14_105854360_C_G	rs549809721	IGHM	0	0	0	0	0.0005056
14_105854618_C_T	rs782070553	IGHM	0	0	0	0.00188	0
14_105855558_C_T	rs1059216	IGHM	0.4224	0.4062	0.2625	0.3797	0.4066
14_105856143_C_T	rs372261472	IGHM	0	0	0	0	0.003033
1_234607828_T_C	rs1012466215	IRF2BP2	0	0.004237	0	0	0
1_234608450_C_T	rs1052817071	IRF2BP2	0	0	0	0	0.000495
1_234609136_G_A	rs1195336113	IRF2BP2	0	0	0	0	0.0005045
1_234608663_C_G	rs1360414372	IRF2BP2	0	0	0	0	0.000495
1_234608614_C_G	rs755757862	IRF2BP2	0	0	0	0	0.000495
4_102610685_G_T	rs1578834273	NFKB1	0	0	0	0.005597	0
4_102613462_T_C	rs200451929	NFKB1	0	0	0	0	0.000495
10_102403342_G_A	rs572223815	NFKB2	0	0.008475	0	0	0
10_102405018_T_C	rs200053412	NFKB2	0	0.004237	0	0	0
10_102405248_G_A	rs759227476	NFKB2	0	0	0	0	0.000495
10_102405451_T_G	rs2135450097	NFKB2	0	0	0	0	0.000495
10_102405463_C_T	rs752053696	NFKB2	0	0	0	0	0.000495
5_68295452_A_G	rs1218229603	PIK3R1	0	0	0	0	0.000495
5_68293360_C_T	rs3730090	PIK3R1	0.06154	0.1864	0.0875	0.1269	0.1322
11_36573364_C_T	rs138801620	RAG1, RAG2	0	0	0	0.01306	0.000495
11_36573401_T_A	rs572993073	RAG1, RAG2	0	0.004237	0	0	0
11_36574837_G_T	rs533793542	RAG1, RAG2	0	0	0	0	0.000495
11_36575125_C_T	rs183806098	RAG1, RAG2	0	0	0	0.001866	0
11_36575763_A_G	rs2227973	RAG1, RAG2	0.04615	0.03814	0.2	0.1082	0.09208
11_36593297_G_A	rs112927992	RAG2, RAG1	0	0	0	0.003731	0
11_36594157_C_G	rs1851109380	RAG2, RAG1	0	0	0	0.001866	0
11_36593015_C_T	rs1157290414	RAG2, RAG1	0	0	0	0	0.000495
11_36593979_T_C	rs535374501	RAG2, RAG1	0	0	0	0	0.000495
11_47359926_C_T	rs1370001180	SPI1	0	0	0	0	0.000495
11_47359927_G_C	rs537934199	SPI1	0.007692	0	0	0	0
19_1646390_G_A	rs1340859555	TCF3	0	0	0	0	0.000495
19_1615692_C_T	rs141432924	TCF3	0	0	0	0	0.00297
19_1611790_C_G	rs146047446	TCF3	0	0	0	0	0.000495
19_1632351_G_C	rs1487181959	TCF3	0	0	0	0	0.000495
19_1622220_C_A	rs2146137895	TCF3	0	0	0	0	0.000495
19_1621889_C_T	rs374898725	TCF3	0	0	0	0	0.000495
19_1621865_G_A	rs531068508	TCF3	0	0	0	0	0.000495
19_1619348_G_T	rs532539524	TCF3	0	0	0	0	0.000495
19_1619800_C_T	rs535113552	TCF3	0	0	0	0	0.000495
19_1611813_C_T	rs538013937	TCF3	0	0	0	0	0.000495
19_1619351_C_T	rs1052692	TCF3	0.09231	0.0678	0.0875	0.0541	0.0505
19_1619793_C_T	rs117006898	TCF3	0	0	0	0.001866	0
19_1620976_C_T	rs148928381	TCF3	0.007692	0	0	0	0.00198
19_1615376_T_C	rs149166972	TCF3	0	0	0	0	0.007921
22_41925453_G_A	rs1303856630	TNFRSF13C	0	0	0	0	0.000495

## Discussion

Genetic diagnosis is increasing in importance in IEIs although in certain populations, genetic information regarding genes associated with disease is incomplete. IEIs are predicted to be higher in prevalence than the numbers included within the registries^17^. This project examined the variation of genes associated with humoral IEIs within an African population through an analysis of the AGVD, a database housing genotype frequency information from countries across Africa collected by projects of the H3Africa consortium. Africa has high genetic diversity with a corresponding paucity of clinical genetics facilities^9,25^ and for this reason, there is a lack of genetic information from African populations regarding humoral IEIs and IEIs in general^17^. We found 815 gene variants within regions of interest for humoral IEI of which 335 were present in individuals of African descent and 219 were only found within African individuals.

### Gene function

The gene variants identified are associated with all aspects of B cell function including B cell development and signalling (CD19, CD79A, CD79B, MS4A1, PIK3R1, IGHM, IGLL1, TCF3 and SPI1), class switch and somatic hypermutation (AICDA and UNG). B-cell co-stimulation and survival (CD40, ICOS, CTLA4, TNFRSF13B, TNFRSF13C, CD81 and CR2) and immune regulation and transcription factors (NFKB1, NFKB2 and IRF2BP2). We also found variants in the RAG1 and 2 genes that could affect V(D) J recombination (RAG1 and RAG2) and hence potentially impact both T-cell and B-cell receptor rearrangement. A comprehensive analysis of genes impacting T-cell development was outside the scope of this study but represents an important consideration when developing diagnostic strategies for IEIs.

### Pathogenic gene variants

The AGVD provides access to genotype frequency information mainly from participants used as controls in different studies and therefore defined as ‘healthy’. Several of the variants described here were classified as benign although some variants were classified as either of uncertain significance or conflicting significance. Unexpectedly, four variants were detected that were classified as pathogenic or likely pathogenic. Several factors may have influenced the clinical presentation of these variants in ostensibly healthy individuals, including the inheritance pattern of the gene (dominant or recessive), penetrance, mosaicism and homozygosity for the deleterious variant. Environmental factors may also ameliorate clinical presentation of inborn errors of immunity^26^. Considering the variable presentation of humoral IEIs^27^, it is possible that the H3Africa criteria for healthy individuals may have included some affected individuals. In one of the variants in the TCF3 gene, a stop-gain mutation was identified, which was predicted to lead to loss-of-function (LOF), an established cause of agammaglobulinemia. Biallelic LOF and monoallelic dominant-negative LOF result in disease, but there are cases of monoallelic loss-of-function resulting in milder forms of immunodeficiency with increased susceptibility to infections^28–30^. RAG 1 and RAG 2 proteins mediate the V(D)J recombination process in both T and B cells^31^ and LOF is associated with severe combined immunodeficiency (SCID) with T-cell and B-cell dysfunction^10^. Three pathogenic or likely pathogenic gene variants were identified in this gene.

### Variants not in ClinVar

Several variants were identified that were not recorded in ClinVar. To assess their potential impact, SIFT and PolyPhen predictions were considered alongside their molecular consequences. Among the variants absent from ClinVar, two stop-gain mutations were identified in the *CR2* and *CD79A* genes, both variants were predicted to be deleterious and possibly damaging by SIFT and PolyPhen. *CR2* encodes a key receptor involved in immune function, particularly in Epstein–Barr virus interactions, and its deficiency has been associated with CVID, which is characterised by decreased serum IgG levels accompanied by a reduction in either IgA or IgM^32^.Ideally, the pathogenicity of this variant should be assessed in functional validation. *CR2* mutations in CVID exhibit an autosomal recessive inheritance pattern, suggesting that pathogenic variants may be present in healthy individuals. CD79A forms part of the B cell antigen receptor; mutations in this gene have been linked to autosomal recessive agammaglobulinemia, which have been shown to be more severe than the X-linked form.

### High frequency variants

The frequencies of variants varied both intra and inter-regionally. The majority of the variants found were rare (genotype frequency less than 0.001) however, several variants predicted to be deleterious by SIFT or PolyPhen-2 were more common (genotype frequencies up to 0.7). Variant effect predicting tools such as SIFT and PolyPhen-2 predict whether the variant will impact protein function or not, but may not necessarily have a negative impact. Variants with higher frequencies in a population (often above 5%) are often either beneficial (adaptive) or neutral^33^. We observed a variant from the CR2 gene (not recorded in ClinVar) which was predicted to have a deleterious effect by SIFT and PolyPhen-2. This specific variant had higher genotype frequencies in several African populations, 0.67 in Central African populations and 0.54 in Western and Eastern African populations. CR2 or CD21 complexes with CD19, CD81 and CD225 to form the B cell coreceptor complex, lowering the B cell receptor’s activation threshold^34^. Epstein-Barr Virus (EBV) binds CD21 during B-cell infection because the EBV envelope protein is homologous to C3d^35,36^. It is therefore possible that mutations in this receptor may affect susceptibility to EBV in affected individuals. Africa is one of the regions with significantly high incidences of Burkitt’s lymphoma and is almost always linked to EBV infection^37^. The potential impact of other high-frequency variants predicted to be deleterious remains to be determined, and it would be important to correlate the genotype with the phenotype including levels of the production of functional protein.

### Clinical implications

 Findings of this study have important clinical implications, specifically in the diagnosis and management of IEIs in individuals of African ancestry. The identification of both known and novel variants in genes involved in B-cell development, class-switch recombination, and immune regulation highlights the need to incorporate African genomic data into diagnostic reference frameworks. The presence of pathogenic or likely pathogenic variants in healthy individuals the absence of many potentially clinically relevant African variants in ClinVar suggests that current variant interpretation strategies, largely derived from European-ancestry datasets, may misclassify clinically relevant mutations in African populations. Furthermore, IEI of immunity range in symptomatic presentation and may be asymptomatic and present later in life, but these individuals remain at risk of related complications. This underlines the urgent need to develop population-specific diagnostic gene panels and variant annotation pipelines to enhance diagnostic yield, reduce the burden of variants of uncertain significance (VUS), and avoid false negatives. Our findings underscore the complexity of this task, revealing that many variants present in African populations remain poorly characterized. Moving forward, expanding the analysis to include all genes implicated in humoral IEIs will provide a more comprehensive view of candidate genes for inclusion in diagnostic panels. The detection of potentially adaptive, high-frequency deleterious variants in genes such as CR2 and IGHM may point to evolutionary pressures shaped by regional infectious disease burdens—an insight that could influence future research into immune resilience and vaccine response. Integrating these findings into clinical practice could significantly improve early detection, genetic counselling, and personalized treatment strategies for IEIs across diverse African populations.

## Limitations

The findings presented in this study have limited clinical applicability, as the data came from healthy participants. Despite this limitation, these data provide valuable insight that can inform the design of future clinical studies. Importantly, the data reported here is a the population level rather than the individual levels. This restricts the ability to track specific variants and determine their zygosity (i.e. whether individuals were homozygous or heterozygous for a gene variant). Furthermore, the study included only 23 genes, whereas more that 60 genes have been implicated in humoral IEIs (Table 1).

## Conclusions

The substantial genetic diversity found in African populations is highlighted in this study, along with its connection to humoral inborn errors of immunity (IEIs). We found 815 gene variants through AGVD analysis, including 335 present in individuals of African descent. Many of these variants impact important genes involved in immune regulation, signalling, and B-cell development. Unexpectedly, a number of variants that were deemed pathogenic or likely pathogenic were discovered in apparently healthy people, possibly indicating variable penetrance, recessive inheritance, or environmental modulation. Several variants not recorded in ClinVar were predicted to have potential functional impact and, in some cases, occurred at genotype frequencies similar to known pathogenic variants. These observations warrant further investigation to clarify their clinical relevance. These findings highlight the significance of using African genomic data in variant interpretation and the requirement for population-specific methods in the diagnosis of IEIs.

## Supplementary Information

Below is the link to the electronic supplementary material.

Supplementary Material 1

Supplementary Material 2

## Data Availability

The data analysed in this study are available from the AGVD (https://nyame.h3abionet.org/accounts/login/?next=/refresh). Access to the data requires the creation of a user account on the AGVD website.

## Abbreviations

IEIs: Inborn errors of immunity
CVID: Common variable immunodeficiency
BTK: Brutton’s kinase
AGVD: African genome variation database
VEP: Variant effect predictor
ACMG: American College of Medical Genetics and Genomics
AMP: Association of pathology
VUS: Variant of uncertain significance
SIFT: Sorting intolerance from tolerance
EBV: Epstein barr virus
